# Inflammatory myofibroblastic tumor of the kidney with viral hepatitis B and trauma: A case report

**DOI:** 10.3892/ol.2013.1598

**Published:** 2013-10-01

**Authors:** ZEYU LI, WEIGANG WANG, YUANTAO WANG, XIUYU ZHAI, YE TIAN, YAOWEN FU, HONGLAN ZHOU

**Affiliations:** 1 Center of Urology, First Hospital, Jilin University, Changchun, Jilin 130021, P.R. China; 2 Department of General Surgery, First Affiliated Hospital, Dalian Medical University, Dalian, Liaoning 116011, P.R. China

**Keywords:** inflammatory myofibroblastic tumor, kidney, viral hepatitis B, trauma

## Abstract

Inflammatory myofibroblastic tumor (IMT) is a rare entity that most commonly involves the lung. However, an IMT of the kidney is extremely rare. The etiology and pathogenesis of IMT remain unknown. The present study describes the case of a 48-year-old female who presented asymptomatically. Imaging investigations revealed a mass in the left kidney and a pathological examination of the nephrectomy specimen revealed an IMT. The patient had a history of trauma in the left hypochondrium 13 years previously and a history of hepatitis B for 20 years. The latter developed into hepatic cirrhosis, hypersplenism and coagulation disorders, which may play have played a significant role in the development of the IMT of the kidney in the present case and also may aid in improving the understanding of the etiology and pathogenesis of IMT of the kidney.

## Introduction

Inflammatory myofibroblastic tumor (IMT) is an uncommon tumor that was first described in the lung and has now been identified at multiple extrapulmonary anatomical sites (1). In the genito-urinary tract, IMT most commonly occurs in the bladder. However, a small series of renal IMT cases have been reported in the medical literature, to date (2–5). The etiology and pathogenesis of IMT remain uncertain; however, infection, vascular causes, autoimmune disorders and the anaplastic lymphoma kinase (ALK) gene have been proposed (7,8). The present study describes a case of a 48-year-old female with an IMT of the kidney, who was treated by radical nephrectomy and had a history of trauma to the left hypochondrium 13 years previously. The patient also had a history of hepatitis B for 20 years, which developed into hepatic cirrhosis, hypersplenism and coagulation disorders and may improve the understanding of the etiology and pathogenesis of renal IMT. Written informed consent was obtained from the patient.

## Case report

**Figure 1:**
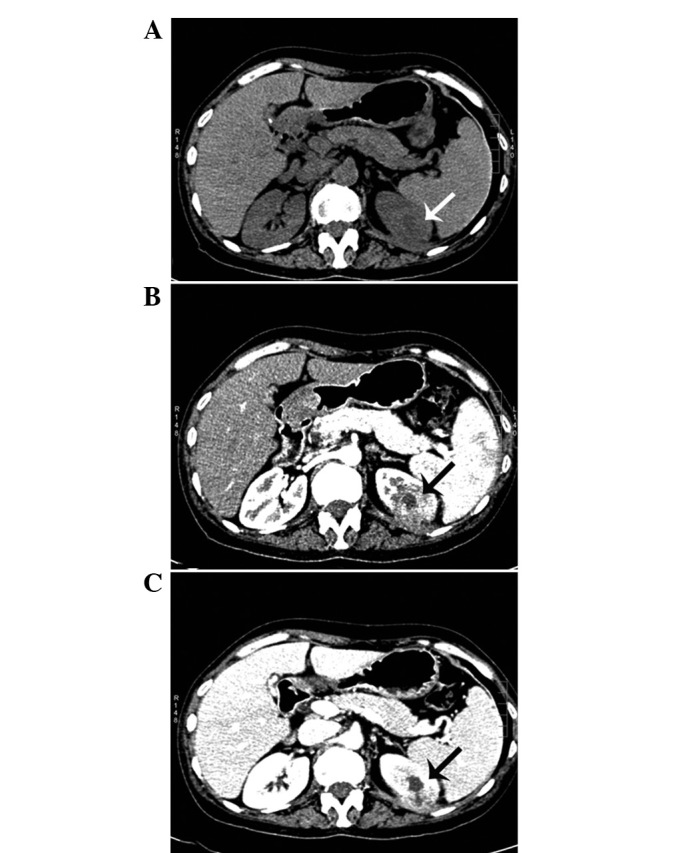
(A) Computed tomography (CT) scan showing a 1.6×2.9×2.0-cm lesion in the upper pole of the kidney (arrow). (B and C) Contrast-enhanced CT scans showing marginal enhancement (arrow).

**Figure 2:**
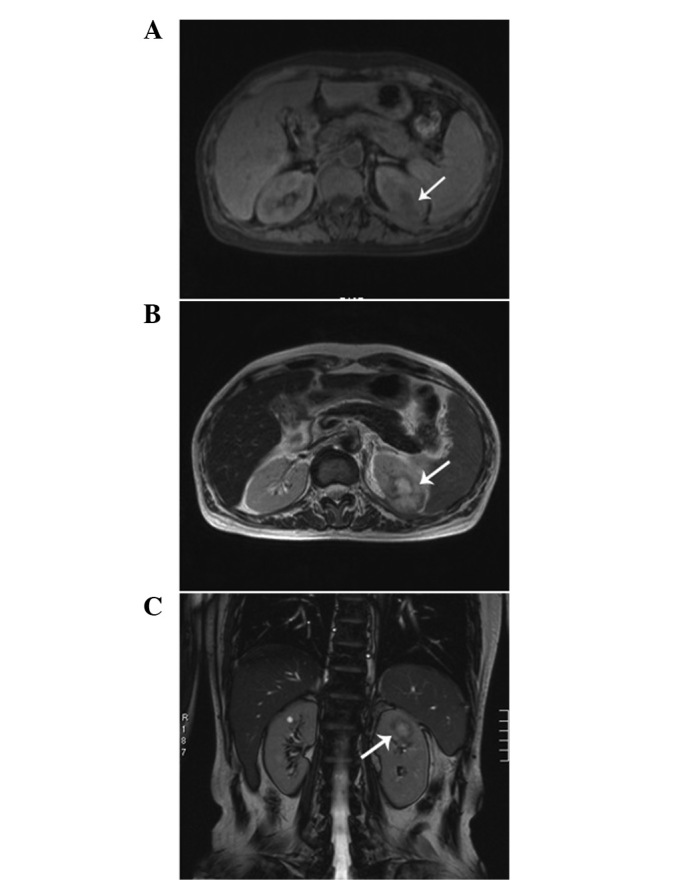
Magnetic resonance imaging demonstrating a heterogeneous mass measuring 2.6 cm. (A) Low intensity on T1-weighted images (arrow). (B and C) High intensity, accompanied by hypointensity surrounding the center of the lesion, on T2-weighted images (arrow).

**Figure 3:**
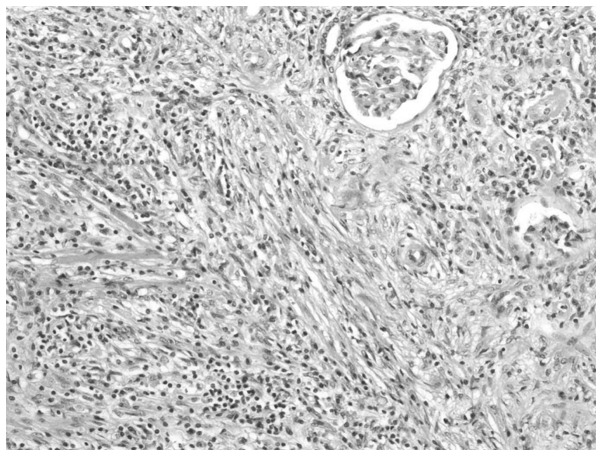
Photomicrograph showing bands of spindled fibroblast-like cells and collagen with infiltrating lymphocytes and plasma cells (HE staining; magnification, ×200).

**Figure 4:**
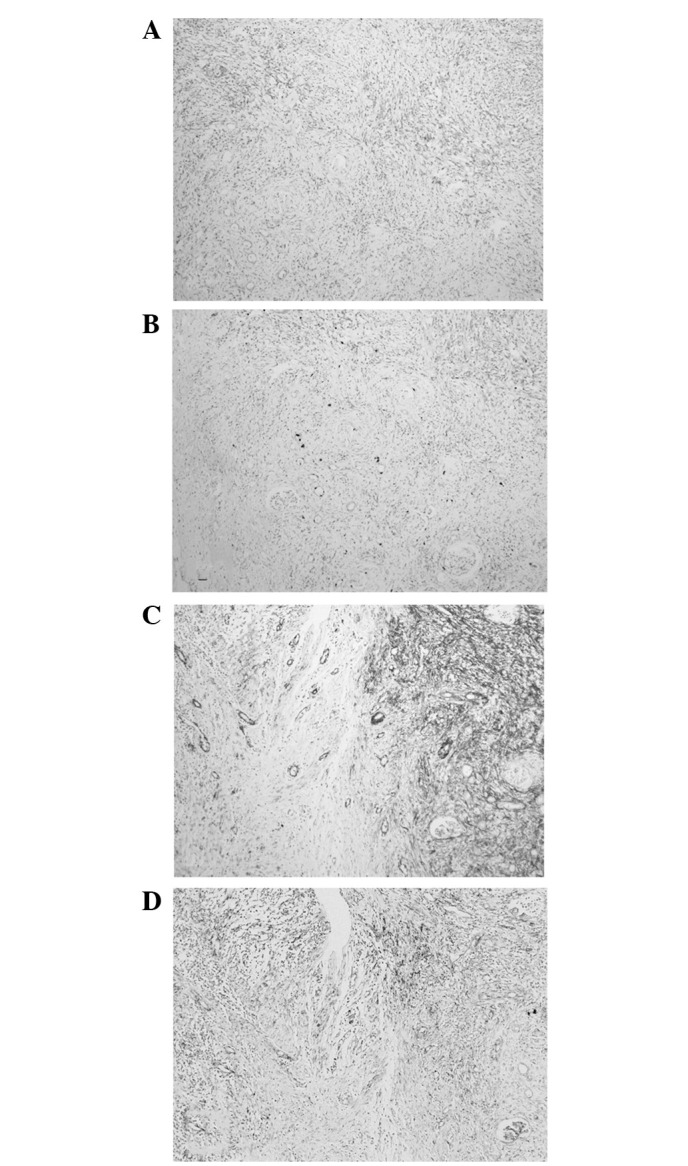
(A) Immunohistochemistry for (A) desmin,(B) Ki-67, (C) smooth muscle actin and (D) vimentin (Avidin-Biotin staining; magnification, ×100)

A 48-year-old female visited the First Hospital, Jilin University (Jilin, China) for routine check-up for hepatitis B on July 19, 2012 and presented with no symptoms. The patient had a history of trauma to the left hypochondrium 13 years previously and a history of hepatitis B for 20 years. The latter developed into hepatic cirrhosis, hypersplenism and coagulation disorders. The physical and basic paraclinical examinations were normal. Blood tests revealed a leukocyte count of 2850/mm^3^, a hemoglobin count of 5.6 g/dl, a platelet count of 6700/mm^3^, a urine leukocyte count of 20.1/HPF and a urine erythrocyte count of 2.5/HPF. The thrombin time was 19.4 sec and the prothrombin time was 14.0 sec. The international normalized ratio was 1.21, the prothrombin ratio was 1.22 and the prothrombin activity was 69%. Clinical laboratory measurments revealed the following levels: Serum fibrinogen, 0.55 g/l; hepatitis B virus surface antigen (HBsAg), 197.260 IU/Ml; hepatitis B virus e antigen, 0.299S/CO; hepatitis B virus e antibody, 0.110S/CO; and hepatitis B virus core antibody, 18.210S/CO. An abdominal ultrasonography revealed a 1.4×1.4-cm-sized mass with an obscure boundary in the upper pole of the kidney, which protruded through the surface. The computed tomography (CT) scan revealed a 1.6×2.9×2.0-cm lesion in the upper pole of the kidney. The CT was slightly enhanced with contrast (Fig. 1). The magnetic resonance imaging revealed a heterogeneous mass measuring 2.6 cm, showing low intensity on the T1-weighted images and high intensity on the T2-weighted images, which was accompanied with hypointensity that surrounded the center of the lesion (Fig. 2). A radical nephrectomy was performed. The histopathological examination resulted in the lesion being diagnosed as an IMT, in which spindle cells were admixed with variable amounts of extracellular collagen, lymphocytes and plasma cells (Fig. 3). Immunostaining was positive for vimentin and focally positive for smooth muscle actin, desmin and Ki-67 (Fig. 4). There was no evidence of recurrence during a follow-up period of six months.

## Discussion

IMT has been recognized as an inflammatory pseudotumor and has been considered to be a reparative post-inflammatory condition rather than a neoplastic process (6). However, rearrangements involving ALK have been documented in pulmonary and extrapulmonary IMTs, supporting the contention that IMT is a neoplasm (7). No definitive cause of the condition has been identified and the etiology is likely to be multifactorial, including infection, vascular causes and autoimmune disorders (8). However, the identification of the ALK gene fusion has allowed an improved recognition and understanding of the pathogenesis of IMT with regard to the genetic aspect (7). Granulomas and giant cells have been observed in the tumor tissue, suggesting that infection is associated with IMT (9). Numerous studies have indicated that IMT may be due to an intraparenchymal hemorrhage that is secondary to trauma or coagulopathy (10). Cotelingam and Jaffe have suggested that the initial event may be a focal parenchymal necrosis with hemorrhage (11). In the present study, the inflammatory reaction was suspected to have occurred as a consequence of the sedimentation of HBsAg or an antigen-antibody complex in the kidney. Furthermore, the intraparenchymal hemorrhage of the kidney may have been due to the left hypochondrium trauma history and the coagulation disorders that were caused by hepatic cirrhosis.

An IMT patient may be clinically asymptomatic or may present with flank pain, painless hematuria and ureteropelvic junction stenosis with hydronephrosis (12). Histologically, IMTs are characterized by a mix of inflammatory cells, including plasma cells, lymphocytes, eosinophils and bland spindle cells without nuclear atypia. The tumors may exhibit necrosis, hemorrhage, focal calcification and mitotic activity (6). Confirming a pre-operative diagnosis is difficult and the tumor may be misdiagnosed as renal cell carcinoma, as the symptoms and imaging findings are not specific and are particularly difficult to distinguish from renal cell carcinoma. Therefore, a nephrectomy is usually selected as a treatment strategy (13). Histological examination is of particular significance to obtain a clear diagnosis. However, an inadequate amount of tissue is often obtained by CT-guided fine needle aspiration. Therefore, the definitive diagnosis is often by histopathological examination of the resected specimen following surgery (14). IMT exhibits a good prognosis; Kapusta *et al* have reported a series of 12 cases of IMT of the kidney, of which the follow-up information was available in eight cases and ranged from 1 to 17 years, with no evidence of recurrence (12).

In summary, IMT of the kidney is extremely rare and the etiology and pathogenesis remain unclear. In the present case, the patient had a history of trauma of the left hypochondrium and a long-term history of hepatitis B, which developed into hepatic cirrhosis, hypersplenism and coagulation disorders. The subsequent conditions may have played a significant role in the development of IMT of the kidney in the present case, and may also be useful in increasing the understanding of the etiology and pathogenesis of IMT of the kidney.
